# A mixed-method analysis of health literacy and indicators of well-being in women with polycystic ovary syndrome across the lifespan

**DOI:** 10.1177/26334941261426089

**Published:** 2026-02-22

**Authors:** Crystal C. Douglas, Ahmar Hashmi, Alexis Mclain, Emily J. Arentson-Lantz

**Affiliations:** Department of Nutrition Sciences and Health Behavior, University of Texas Medical Branch, Galveston, TX, USA; Center for Health Promotion, Performance, and Rehabilitation Research, University of Texas Medical Branch, Galveston, TX, USA; Department of Nutrition Sciences and Health Behavior, University of Texas Medical Branch, Galveston, TX, USA; Dissemination and Implementation Core, Greater Gulf Coast Clinical Translational Science Alliance, University of Texas Medical Branch, Galveston, TX, USA; Department of Nutrition Sciences and Health Behavior, University of Texas Medical Branch, Galveston, TX, USA; Department of Nutrition Sciences and Health Behavior, University of Texas Medical Branch, Galveston, TX 77555-1124, USA; Center for Health Promotion, Performance, and Rehabilitation Research, University of Texas Medical Branch, Galveston, TX, USA

**Keywords:** aging, female, hyperandrogenism, menopause, PCOS

## Abstract

**Background:**

Despite the severe, chronic nature of polycystic ovary syndrome (PCOS), relatively little is known about the lived experience of women with this condition, especially as they transition through menopause.

**Objective:**

This mixed-methods study investigated the lived experience of women with PCOS before and after the menopause transition to understand their health literacy, barriers to healthcare management, and desired resources to improve their health and well-being.

**Design:**

This was a convergent-parallel mixed-methods study.

**Methods:**

Twenty-four participants completed semi-structured interviews and electronic surveys between April 2023 and August 2024. Qualitative data were analyzed using an inductive open-ended approach for thematic analysis.

**Results:**

Participants, including 17 pre-menopausal (30.1 ± 4.8 years) and 7 post-menopausal (58.6 ± 6.0 years), self-reported clinical symptoms of PCOS (irregular cycles, hirsutism, and acne) and higher than average anxiety symptoms (pre-menopausal only). Both pre- and post-menopausal women were knowledgeable about the impact of PCOS on their fertility, and expressed low to moderate health literacy to successfully manage the PCOS-related symptoms. Few participants expressed understanding of long-term chronic disease risk. Pre-menopausal participants sought resources for managing symptoms but reported dissatisfaction with provider education and overall patient care. Post-menopausal participants did not view a PCOS diagnosis as a health concern following menopause and internalized PCOS-related health issues as something to be endured. Both pre- and post-menopausal women expressed desires for improved personalized care, life-stage-specific support groups, and better patient-facing resources.

**Conclusion:**

Pre-menopausal and post-menopausal women with PCOS exhibit low health literacy about the potential impact of PCOS on metabolic health. Primary care providers should be trained in how to educate women with PCOS, with an emphasis on the impact of the disease beyond reproductive health and through the lifespan. In addition, creating patient-centered resources supporting women throughout the lifespan is needed.

**Trial registration:**

This study was registered with ClinicalTrials.gov as NCT05769426.

## Plain language summary

Well being and health knowledge in women with PCOS before and after menopause

Polycystic ovary syndrome (PCOS) is a serious, long-lasting condition, but we don’t know much about what women with PCOS go through, especially during menopause. This study looked at women’s experiences with PCOS before and after menopause to understand: 1) What they know about their condition, 2) What makes healthcare difficult and 3) What resources they need. We talked to 24 women and had them fill out surveys. We carefully analyzed their answers to find common themes. We studied 17 younger women who hadn’t gone through menopause yet and 7 older women who had. Both groups reported typical PCOS symptoms like irregular periods, extra body hair, and acne. Younger women showed higher anxiety levels. All women knew how PCOS affected their ability to have children but weren’t confident in managing their symptoms. Few understood that PCOS increases risk for other health problems. Younger women looked for help with symptoms but were unhappy with their doctors’ knowledge and care. Older women didn’t think PCOS mattered after menopause and believed they just had to live with their health issues. Both groups wanted better personalized care, support groups for their age, and helpful resources. Women with PCOS don’t know enough about how it affects their overall health. Doctors need better training to explain PCOS effects beyond just reproductive issues. We also need to create better resources to help women with PCOS throughout their lives.

## Introduction

Polycystic ovary syndrome (PCOS) is a chronic endocrine disorder affecting up to 20% of women.^1^ Diagnostic guidelines have evolved,^2^ but the most widely accepted criteria include the presence of two or more of the following features: irregular menses, presence of biochemical or clinical hyperandrogenism (often manifested in women with PCOS as hirsutism), or polycystic ovarian morphology.^3,4^ Consequently, four phenotypes of PCOS exist; phenotype A includes all three diagnostic criteria, and phenotypes B, C, and D include a different combination of two criteria.^3,4^ This complex and heterogeneous condition is also associated with dermatological, endocrinological, cardiometabolic (diabetes, obesity, and insulin resistance), and psychological (anxiety and depression) comorbidities.^2,5,6^ Longitudinal community-based cohort studies, such as the Study of Women’s Health Across the Nation, have elucidated the deteriorating impact of aging on health measures in women across the menopause transition.^7–9^ However, studies assessing the long-term health outcomes in women with PCOS are limited^10^ although there is some evidence to suggest that some comorbidities persist.

PCOS influences physical, emotional, and social aspects of patients’ lives and often contributes to a decline in health-related quality of life. Specifically, reproductive-aged women with PCOS report impaired life satisfaction related to hirsutism, increased body weight, irregular menses, and related infertility.^11,12^ The common health concerns related to PCOS, such as irregular menses and infertility, cease with menopause, but other factors that influence life satisfaction, such as body weight and hirsutism, persist. Extensive literature exists describing increased health risks^13^ and declining quality of life^14^ among women following the menopause transition,^15^ but there is a paucity of evidence evaluating the quality-of-life indicators and lived experiences of post-menopausal women with PCOS.

First line of lifestyle management of PCOS prioritizes a comprehensive approach emphasizing weight management through dietary interventions, increasing physical activity, stress management, health risk reduction, and symptom management.^2,6,16–18^ These strategies primarily target reducing the chronic disease risk associated with PCOS but may also improve quality of life. Implementation of these strategies requires a relatively high level of health literacy to incorporate the information from health care providers and make appropriate health-related decisions.^19^ To date, lifestyle interventions in reproductive-aged women with PCOS are minimally effective and difficult to successfully adopt long term.^20^ Women with PCOS often display low health literacy, which is a barrier to successful management of any chronic disease, including PCOS. Low health literacy in women with PCOS manifests as poor nutrition knowledge^21^ and self-directed dietary choices,^21,22^ disordered eating^23,24^ and increased sedentary time.^25^ An additional barrier to successful management reported by health care providers is the heterogeneity of the condition^20,26^ and the patients have described uneducated providers and one-size-fits-all lifestyle interventions.^26–28^ Little is known regarding the health literacy of post-menopausal women with PCOS or how they incorporate information from their health care provider to manage their health as they age.^29,30^

The objective of this study was to explore and contrast the lived experience of women with PCOS across the lifespan with an emphasis on quality of life and health literacy.

## Methods

We used a convergent-parallel mixed-methods design to explore the lived experience of women with PCOS across the lifespan. Quantitative data and qualitative data were collected concurrently between April 2023 and July 2024. Quantitative data included demographics, quality-of-life measures, and self-reported PCOS clinical presentation, including hirsutism scores. Qualitative data were collected through semi-structured interviews conducted with participants following quantitative data collection. This study was conducted and reported in accordance with the COREQ statement.^31^ A COREQ checklist is included as Supplemental File 1.

### Participants

Adult females (*n* = 24) were recruited from the greater Galveston/Houston, TX area utilizing digital advertising, flyers, and provider referrals. Discrete age cut-offs were selected to highlight comparisons between two groups: pre- and post-menopausal women. Perimenopause is a transitional phase that begins up to 10 years prior to a woman experiencing menopause, which occurs at approximately 51 years in US women.^32^ Thus, eligible participants were ages 18–40 (pre-menopausal; PRE) and ⩾50 years (post-menopausal; POST), with a body mass index between 18.5–40 kg/m^2^ (POST only) and self-reported having PCOS. All participants reported a medical diagnosis of PCOS by a healthcare provider; however, we were unable to verify this using medical records. Menopausal status was self-reported as no menses in the previous 12 months, in accordance with STRAW criteria.^33^ Exclusion criteria included women who were currently pregnant, breastfeeding, or reported surgical or pharmacologically induced menopause (e.g., hysterectomy, oophorectomy, or chemically induced menopause). All participants underwent a telephone screening to confirm eligibility. If participants were eligible and indicated interest in participating, study details were presented during the phone call, and electronically captured written informed consent was provided through the REDCap consent module. All participants received monetary compensation for their time and effort.^34^

### Quality-of-life indicators

Participants completed several electronically administered questionnaires (REDCap electronic data capture tool hosted by UTMB) to answer questions about quality-of-life indicators. NIH PROMIS Depression v8a and PROMIS Anxiety v8a measures were used to assess depression and anxiety symptoms.^35,36^ Participants also self-reported medication usage to manage anxiety and/or depression. NIH PROMIS Ability to Participate in Social Roles v2.0 4a and Activities and PROMIS Satisfaction with Social Roles and Activities v2.0 4a measures were used to assess social function by asking participants to report perceived ability and satisfaction to perform usual social roles and activities.^37^ For the PROMIS outcomes reported here, a score of 50 is average for the United States population; a score of 10 less than or higher than 50 (e.g., 40 or 60) is considered to be lower or higher than average.

### Clinical presentation of PCOS and hirsutism

Participants completed an adapted screening questionnaire from Bedrick et al.^38^ to evaluate and quantify the clinical presentation of PCOS. Hirsutism is frequently evaluated using the modified Ferriman Gallwey (mFG) system, comprised of 9 body sites (upper lip, chin, chest, upper and lower back, upper and lower abdomen, arm, and thigh) with scores of 0–4 (no hair up to severe hair present) possible for each site.^39^ Subsequent developments, including the graphical tool for scoring^40^ and determination of cut-off values defining hirsutism (score ⩾8)^41^ have eased the assessment process for the medical provider, though population characteristics and observer bias may yield variable results. For this protocol, hirsutism was self-evaluated using a simplified Ferriman Gallwey (sFG) scoring system comprised of six body sites (upper lip, chin, chest, upper and lower abdomen, and thigh), including areas sensitive to the effects of androgens.^42^ The total score was calculated, and an sFG score of ⩾8 was considered to indicate the presence of hirsutism.^41^ Participants were also asked about hair removal techniques, menstrual length and regularity, and the presence of acne. Post-menopausal participants were asked to complete the screener by recalling their hirsutism severity and clinical presentation prior to menopause.

### Semi-structured interviews

This study was conceived by C.C.D. and E.J.A.-L. to describe the lived experience and health literacy of younger (pre-menopausal) women and older (post-menopausal) women with PCOS. Both researchers have a shared interest in women’s health as well as individual expertise in PCOS (C.C.D.) and aging (E.J.A.-L.). We wondered how aging may affect women’s perspectives of their diagnosis and what health knowledge tools they accessed to manage their health. C.C.D. and E.J.A.-L. included A.H. as an investigator, given his expertise in qualitative research and as a trained medical doctor. C.C.D., E.J.A.-L., and A.H. had no prior engagement with participants or were involved in any aspect of their patient care.

To limit researcher influence on interviewer-participant dynamics and to help prevent social desirability bias, all interviews were conducted by two trained master’s-level female research staff not involved in participants’ care. The interviewer contacted the participant to schedule a time that worked for them to be interviewed using a video conferencing platform. Before beginning the interview, the participants were reminded of the purpose and content of the study, reminded that the interview would be recorded and stored on a secure server, and that they could stop at any time or not answer a question if they were uncomfortable. A semi-structured guide was used and consisted of eight open-ended questions regarding participant experiences with PCOS (Supplemental Table 1). Specifically, questions were designed to assess topics including diagnosis, barriers, management practices, resources, and self-efficacy. The open-ended nature of the questions allowed participants to describe their own experiences at will. The interview guide was developed based on the work of Copp et al.^26^ and expanded by the research team (C.C.D. and E.J.A.-L.) to explore participant self-efficacy in managing their health and desired resources. Periodically, the researchers expanded the conversation by asking additional, unscripted questions. These clarifying and supplementary questions aimed to obtain further insights regarding the participant’s experience with PCOS, including experiences with medical providers or diagnostic challenges. At the end of the interview, the interviewer expressed gratitude for the participant’s contribution to the study.

Participant interviews lasted approximately 20–60 min and were conducted from April 2023 through July 2024. Data saturation was determined by consensus between C.C.D. and E.J.A.-L., following the summarization of main themes and concepts emerging from each interview, and deciding if further interviews were needed to uncover more information from participants. At the end of the interviews, no significant new qualitative information was encountered, and the dataset was considered complete. All interviews were transcribed verbatim using NVivo v15 (Lumivero, Denver, CO, USA) software, and all original transcribed documents were cross-referenced with recordings from each interview to ensure accuracy. Any inaccuracies from the original transcription were corrected to match the Zoom or phone call recording by study staff.

### Data analysis

Interviews were analyzed using a constructivist approach that assumed that there is more than one reality based on the participants’ experiences with PCOS.^43–45^ Thematic analysis followed an open-ended, inductive approach.^46^ C.C.D. and E.J.A.-L. created an analysis process that began with open coding of two transcripts. C.C.D., E.A.L., and A.H. convened to generate consensus on emergent codes and created a codebook with definitions to guide coding the remaining transcripts. In-person consensus meetings were held regularly to ensure that coding was similar across researchers, with each researcher providing their input, followed by a discussion where each researcher provided their viewpoint, and then an agreement was reached. A final meeting (C.C.D., E.J.A.-L., A.H.) to determine themes was then performed, with an eye to corroborating, triangulating, and explaining the quantitative components of the study.^43–46^

### Statistical analysis

Group differences of the descriptive measures (age of diagnosis, quality of life indicators) were determined via a two-tailed t-test with statistical significance set at *p* < 0.05.

## Results

### Sample characteristics

A total of 24 participants (PRE, *n* = 17; POST, *n* = 7) completed the interviews and questionnaires about PCOS and quality-of-life indicators. Participant demographics are included in Table 1.

**Table 1.** Participant demographic and socioeconomic characteristics.

Socio-demographic characteristics	PRE^a^	POST^b^
Age (years)	30.1 ± 4.8	58.6 ± 6.0
Race (*n*, %)	12, 70.5% White 1, 5.9% Asian 3,17.6% Black or African American 1, 5.9% More than one race	7, 100% White
Ethnicity (*n*, %)	12, 70.6% Not Hispanic or Latino 5 29.4% Hispanic or Latino	7, 100% Not Hispanic or Latino
Household income (*n*, %)		
Less than $10,000	1, 5.9%	0, 0%
$10,000–$24,999	3, 17.6%	1, 14.3%
$25,000–$49,999	2, 11.8%	1, 14.3%
$50,000–$74,999	6, 35.2%	2, 28.6%
$75,000–$99,999	1, 5.9%	0, 0%
$100,000–$149,999	1, 5.9%	2, 28.6%
$150,000 and greater	2, 11.8%	1, 14.3%
Prefer not to answer	1, 5.9%	0, 0%
Employment status (*n*, %)		
Employed for wages	14, 82.3%	6, 85.7%
Student	3, 17.6%	0, 0%
Retired	0, 0%	1, 14.3%

Data is reported as mean ± standard deviation.

a PRE: Pre-menopausal women with PCOS.

b POST: Post-menopausal women with PCOS.

PCOS, polycystic ovary syndrome.

Only 23.5% of pre-menopausal participants reported using hormonal contraceptives (Table 2). An irregular cycle length (<21 days or >35 days) was reported by nearly half of the pre- and post-menopausal groups (46.2% vs 57%; data not shown). While mean sFG (hirsutism) scores were similar between groups (Table 2), nearly 25% of pre-menopausal participants reported scores ⩾15, indicating moderate to severe hirsutism.^39^ Most pre- and post-menopausal participants (88.2% vs 85.7%) reported relying on hair removal techniques (data not shown). Acne was more frequently described by the pre-menopausal participants (pre- vs post-menopausal, 64.7% vs 28.6%; data not shown).

**Table 2.** Hirsutism and quality-of-life indicators.

Hisutism and quality-of-life indicators	PRE^a^	POST^b^	*p*-value
Age of PCOS diagnosis (years)	21.5 ± 4.5	23.6 ± 7.3	0.50
Hirsutism			
Simplified Ferriman Gallwey(sFG) score	10.6 ± 5.9	10.7 ± 4.9	0.74
No hirsutism (sFG score <8; *n*, %)	6, 35.2%	1, 14.2%	
Hirsutism present (sFG score ⩾8; *n*, %)	11, 64.7%	6, 85.7%	
Quality-of-life indicators			
PROMIS anxiety	62.4 ± 2.0	52.0 ± 2.1	**0.012**
PROMIS depression	54.2 ± 1.8	47 ± 2.7	**0.031**
PROMIS ability to participate	49.3 ± 2.8	50.5 ± 2.2	**0.005**
PROMIS social satisfaction	45.1 ± 2.4	55.9 ± 2.5	0.62
Current medication usage			
Hormonal contraception (*n*, %)	4, 23.5%	—	—
Hormone replacement therapy (*n*, %)	—	0, 0%	—
Mood disorder medications (SSRIs/SSNIs; *n*, %)	3, 15.8%	2, 28.6%	—

Data is reported as mean ± standard deviation.

a PRE: Pre-menopausal women with PCOS.

b POST: Post-menopausal women with PCOS.

PCOS, polycystic ovary syndrome; SSNI, serotonin and norepinephrine reuptake inhibitors; SSRI, selective serotonin reuptake inhibitors.

Significant differences (*p* < 0.05) between the pre-menopausal and post-menopausal participants are bolded.

### Quality-of-life indicators

Pre-menopausal participants reported higher than average anxiety symptoms (Table 2), while the post-menopausal group did not, contributing to a significant (*p* = 0.012) difference between the groups. Both groups described average depressive symptoms and social function (Table 2), although there was a significant difference in average scores between the groups (PRE vs POST, PROMIS Depression, *p* = 0.031, and PROMIS Ability to Participate, *p* = 0.005, respectively). However, 15% of pre-menopausal women and 28.5% of post-menopausal women reported using prescription medication to manage mood disorders (Table 2).

### Qualitative analysis

Thematic analysis of the interviews with women with PCOS uncovered three overarching themes: (1) health literacy issues, (2) a desire for customized resources to manage PCOS-related health issues, and (3) dissatisfaction with the patient-provider relationship. Although the themes are distinct, the three themes fundamentally relate to a limited knowledge about PCOS. Pre- and post-menopausal women often had distinct responses to their understanding of their condition.

#### Health literacy issues

Overall, women with PCOS, regardless of menopausal status, demonstrated limited understanding of PCOS beyond a superficial awareness of PCOS as a reproductive disorder. This limited participants’ understanding of PCOS to more than just a constellation of symptoms. A few participants mentioned possible PCOS-related health risks and implications with aging.

Among pre-menopausal women, reports of PCOS-related symptoms were largely focused on hirsutism, weight gain, and irregular menses. Motivation for PCOS management was primarily concentrated on fertility.

My understanding of PCOS is that it is a hormonal problem that is caused by the development but not the full development of ovaries. I mean eggs in the ovary they often will not release and they cause like little cysts on the ovaries and resulting in high levels of often testosterone and irregular periods, difficulty conceiving, hair growth, high levels of insulin and an increased risk of diabetes. – HERBS003, age 37 years

Oh, okay. I honestly don’t know much, but I know it can impact your fertility, which is kind of was my main concern, but I don’t know much if I’m being honest. I remember my doctor being surprised that I had it because I am very active and I’m not heavier. I am very fit, but I don’t know much about it. I should know more, but I don’t. – HERBS002, age 23 years

In the post-menopausal group, participants recounted their experiences receiving a PCOS diagnosis. Participants frequently recalled fertility challenges during their reproductive years, but few women understood the link between their PCOS diagnosis earlier in life and their current health status or future health risks. In fact, several post-menopausal participants reported they were prescribed obesity pharmacotherapy for weight management, but several described stopping medications due to difficulties managing their healthcare expenses. As the following quote suggests, over time, many post-menopausal women merely accepted their condition and its sequelae as a response to their lack of understanding.

Now I feel like it’s under control. I guess. I don’t know. Like, so when somebody goes through menopause, do they still have PCOS or does it even matter if they have cysts on their ovaries if they’re not having a period? You know what I mean? Like, I don’t know. I haven’t even thought that deep into it, really. – HERBS059, age 59 years

#### Desire for resources

This discussion of PCOS knowledge and awareness led to an insightful discussion on the information women seek when uncertain of their condition. Both groups were in agreement about the need for more evidence-based, patient-centered resources, particularly for customized dietary management practices for women with PCOS. All participants appeared to understand that lifestyle modification was inherent to successful PCOS management. Participants frequently expressed a desire for an increased supportive network in the form of PCOS support groups.

The motivation to seek PCOS-related knowledge appeared greater in the pre-menopausal group. For example, pre-menopausal women reported they frequently sought health information online, including social media sites such as Facebook or TikTok.

I tried like mainly some informative, like forums online. Uh, nothing really constant that I checked besides but believe it or not, TikTok is very informational. I went on TikTok often, there’s a lot of girls, surprisingly, that have it. And some of them are like, they look like they’re doing pretty well with it. Um, but it’s harder to kind of follow someone else’s journey when you’re two different people, two different types of bodies. Some are moms, some are not. You know what I mean? Difference in ages[. . .]. So yeah, so it’s just like it’s kind of hard to follow. But yeah, you try to take in some tips here and there, try some different vitamins and probiotics that they recommend. But so far with me, I’ve only had about one or two things that help a little, but it’s about the same. And then, you know, everybody’s taste was different. I don’t really care for their diet. But it is different strokes for different folks, you know what I mean? Yeah so I’m still a work in progress on that, you know. Right. – HERBS001, age 31 years

All the stuff online is like for the layperson, I like it. I would like access to the more detailed things without paywall, the really like, you know, what would a doctor do? What are the treatment guidelines for the situation I’m in? And they’re hard to find. – HERBS003, age 37 years

In contrast to pre-menopausal women, post-menopausal women differed in their access to online resources, where younger, pre-menopausal women had more access to resources over the course of their diagnosis and expressed comfort with online resources more generally than post-menopausal women.

So really how I learned about it and even heard about it the first time is when the doctor said, ‘I know why you’re not getting pregnant. You don’t ovulate.’ And I’m like, ‘Well, why not?’ And then he started talking about PCOS. And then of course, I read more about it. And, you know, back then it wasn’t like the Internet, what we have today. – HERBS059, age 59 years

I wish I knew more about it. More education. I mean, all they would say is, ‘Oh that’s why you have excess hair.’ They never really talked about, you know, - having difficulty with childbirth or anything like that. With pregnancy. – HERBS072, age 57 years

Continuing to gain knowledge so that nobody had to struggle alone like I did. – HERBS061, age 66 years

Regardless of access to information, both groups of women expressed a need for better information, and that this may reduce anxiety felt prior to diagnosis and ongoing management of PCOS.

#### Dissatisfaction with the patient-provider relationship

Knowledge about and information regarding their condition led women to reflect on their relationship with their providers. Implicit in many participants’ comments was that providers were often the primary source of trusted information and advice on PCOS. However, the nature and character of patient-provider relationships were starkly different between the PCOS groups. Pre-menopausal women frequently expressed the desire for more engagement in their health care decisions, frustration with provider support, and a willingness to look elsewhere for better care.

She told me I had PCOS. Um. But me not knowing what that is, I’m like, ‘Well, you know what does that mean?’ She was like, ‘It’s something called polycystic ovarian syndrome. Um, there’s you just get cysts on your ovaries. It doesn’t really do much, but it can cause irregular periods, bloating, things like that.’ But when I look at it online, it’s like it’s much more detailed and all of this other stuff. So I’m just like, ‘Well, I wasn’t born of any of this.’ She told me that once she saw that I had a huge cyst on my ovary so beforehand I wasn’t even aware. – HERBS001, age 31 years

But I also see like a generation of providers willing to learn and to want to be more helpful and not just the written off of eat less and move more because, I mean, that’s just that doesn’t work. – HERBS004, age 33 years

Well, maybe. Sometimes I’m thinking I’m like, maybe the doctor didn’t even have a lot of knowledge of PCOS, so maybe that’s why I wasn’t taken seriously. But I think maybe informing the doctors a bit more [that] PCOS is not the same in every woman. So, seeing the different factors of PCOS or having open minds. ‘Maybe this could be PCOS. We can rule this out before we can continue with the next steps’ – HERBS006, age 26 years

In contrast, the post-menopausal group reported they did not believe medical management was still relevant as they ceased experiencing most PCOS-related symptoms, with the exception of weight gain. As a result, they felt largely unaffected by their PCOS diagnosis after menopause and described greater satisfaction with their patient-provider relationship.

Yeah. I think that I just kind of had to go with the flow because there was really no managing it. It just seemed to. Like, I don’t really have any control over it. I can’t make it stop, you know? But now it’s fine. Now I feel like it’s under control. I guess. I don’t know. Like, so when somebody goes through menopause, do they still have PCOS or does it even matter if they have cysts on their ovaries if they’re not having a period? You know what I mean? Like, I don’t know. I haven’t even thought that deep into it, really. – HERBS059, age 59 years

[PCOS is a. . .] Lifelong issues problem. I’m not as familiar with everything because I about, I want to say about 2010, I just kind of got tired of dealing with. Living my life around PCOS. So, I just kind of backed away from all of that. So I haven’t really even kept up with any of the research or anything. – HERBS071, age 56 years

You know, some of the symptoms are still the same. Some are different. But you know, I’m 54 years old, so I just I’ve learned to deal with it. – HERBS065, age 54 years

The post-menopausal group appeared to have developed different coping mechanisms to cope with the health struggles associated with their PCOS and passively accepted the relationship with their current provider. Whether time to reflect on this prior life stage or perspective with aging was at play, this group of women accepted or internalized prior hardships and made peace with them. This may suggest why quality-of-life indicators for this group, according to PROMIS, showed lower anxiety scores than pre-menopausal women.

In summarizing the descriptive thematic analysis, these three themes—although distinct—were often interrelated. Health literacy issues related to a lack of awareness prior to receiving the diagnosis or with a specific understanding of symptoms as they arise over the course of PCOS. This lack of awareness often led women to seek out or reflect on the desired information about their condition. Participant responses imply that providers are a trusted and respected resource for information and knowledge about PCOS. However, the women expressed dissatisfaction with providers because of their limited engagement in addressing women’s health concerns related to PCOS diagnosis and management. Generational differences between pre- and post-menopausal women help describe how women address issues centered on knowledge of PCOS, the ongoing health issues related to it, and its management.

## Discussion

We employed a convergent-parallel mixed-method approach to examine the lived experience of women with PCOS before and after the menopause transition. While depressive symptoms and social function were not different than average, the pre-menopausal participants reported higher than average anxiety symptoms. Most of the pre-menopausal and post-menopausal participants reported the presence of hirsutism. Qualitative components identified gaps in health literacy, particularly for understanding PCOS beyond fertility challenges, dissatisfaction with healthcare provider education and overall patient care, and the desire for personalized, life-stage–specific resources. Pre-menopausal women with PCOS desired resources for symptom management, while post-menopausal women appeared unaware of a long-term diagnosis of PCOS and related chronic disease risks. Limited awareness of PCOS patient education materials and naivete of the longevity of PCOS and related symptoms were previously identified in the development of a Health Literacy scale for PCOS among a pre-menopausal PCOS population in China.^47^ These findings could guide provider education regarding improved patient-client communication and understanding of the diagnosis and discussion of acute and long-term impacts of the condition and appropriate resources for the life stage.

The increased anxiety symptoms observed among the pre-menopausal participants are not unexpected, as the negative psychological impact of PCOS among reproductive-age women is well described.^48^ Less is known about how mood disorders may persist in women with PCOS following the menopause transition. In a population-based 15-year follow-up, Karjula et al.^49^ compared anxiety and depression symptoms between women with PCOS and controls at ages 31 years and later at 46 years. They reported an increased prevalence of anxiety/depressive symptoms among women with PCOS compared to controls at baseline, and symptoms remained elevated at follow-up,^49^ indicating that mood disorders persist as women approach the menopause transition. Primary care providers report low awareness of mood disorders as a clinical presentation hallmark of PCOS,^50^ which may contribute to a lack of screening for anxiety and depressive symptoms.

The diagnostic criteria for PCOS have evolved over time with greater medical understanding. In addition, technological advancements through social media have made complex medical information widely available to the public. Yet, extensive health misinformation on social media creates a barrier to evidence-based literacy.^51^ Reproductive-aged women with PCOS expressed greater understanding of the health risks associated with PCOS, including concerns for diabetes and cardiovascular disease, which may reflect an increased understanding of the condition or possession of increased technology literacy. According to behavioral theories such as the Health Belief Model, the increased anxiety reported among this group may reflect perceived severity of the condition or perceived barriers to health outcomes. For example, the symptoms of PCOS are most prominent during the reproductive years, and perhaps, the risks of anti-anxiety medications for the pre-menopausal group appear unreasonable and inconsistent with the primary management goal of fertility. Health literacy is a critical skill for women with PCOS to implement behavioral strategies to manage symptoms and mitigate poor health outcomes associated with aging. Perhaps it reflected the time at diagnosis, social or economic inequities, or failure to recall the details of the past, but the post-menopausal group appeared reluctant to ask questions of medical providers concerning their diagnosis. Using a validated health literacy tool in a cross-sectional design study of primarily young and educated Chinese women with a recent diagnosis of PCOS, Liu et al.^52^ described most participants as having limited health literacy, which was positively linked with poor behavioral choices, including diet, physical activity, and stress management. In addition, the authors also observed that increased time since diagnosis of the disease and age were linked with increased health literacy. These findings contradict our conclusions and further support enhanced exploration of the lived experiences of women with PCOS across the lifespan.

Given the different viewpoints on living with PCOS from the sample population, it was not surprising to see contrasting views of the patient-provider relationship. Consistent with the literature, both groups reported gaps in provider knowledge specific to PCOS^53^ and a need for multiple visits with various providers to receive a diagnosis.^6^ Many of the pre-menopausal participants were educated and actively employed in the healthcare sector and likely had higher standards for providers and their care. Furthermore, limited understanding among primary care providers, coupled with higher standards for care, suggests why pre-menopausal women in this study described general dissatisfaction with prioritization of care and knowledge about PCOS. On the contrary, the post-menopausal women seemed reluctant to discuss any current challenges with PCOS, apart from weight gain, a symptom frequently observed among aging women. Whether this omission reflected time since diagnosis or is evidence of their limited understanding of the chronic risks of PCOS is unclear from this study. However, these findings appeared related to the unique demands women expressed for personalized PCOS materials. Similarly, both groups reported seeking informational resources on their own and expressed a desire for more evidence-based patient-centered resources, particularly for diet and lifestyle guidance.^54^ These findings may be more generalizable to self-management of chronic diseases, such as endometriosis, whereas therapy is largely centered around self-care practices. O’Hara et al. described that women diagnosed with endometriosis frequently sought medical information from various resources for support or therapy options and credited a positive patient-provider relationship with enhanced self-management practices and self-efficacy.^54^

### Limitations

The present study is not without limitations. We relied upon participant self-report of PCOS as the diagnostic process is challenging, has evolved, and documentation generated two to three decades beforehand for the post-menopausal group would have been difficult to attain. Though we did ask participants to describe their clinical presentation of PCOS during the pre-menopausal period, we did not investigate which diagnostic criteria were employed or consider PCOS phenotype, an outcome relevant only to those participants diagnosed using the Rotterdam criteria, released in 2003. Hirsutism may have been underestimated, as the cut-off value of ⩾8 was based on hair growth in only six body regions. Retrospective self-report of symptoms, including hirsutism, may provide inaccuracies or introduce recall bias, but, in this case, it served as a practical means to obtain historical, clinically relevant data to support a PCOS diagnosis, particularly for the post-menopausal group. Compared to clinician-report, self-report of hirsutism among women is overestimated^55^; however, Bedrick et al. previously demonstrated the utility of hirsutism self-screening to predict PCOS within a community population.^38^ This was intended to be a hypothesis-generating pilot study, and therefore, the small sample size recruited from an urban area of the southern US may not be representative of all women with PCOS and may not be powered to detect significant differences between the pre-menopausal and post-menopausal women. High educational attainment of this sample may have also influenced outcomes, including health literacy, which was not objectively measured. This may have also influenced the perception of provider education and the nature and character of desired resources. Inclusion of medication use and anxiety measures, though self-reported, supports the distinction of a unique PCOS patient profile across the lifespan. Since PCOS is diagnosed in the reproductive years, it is possible that the experiences of the post-menopausal participants were influenced by the time elapsed. Despite these limitations, all participants passed an initial screener to help support a medical diagnosis of PCOS. In addition, the use of the semi-structured interview enabled us to include predetermined questions for all participants and explore emerging themes of PCOS across the lifespan. Future research should prioritize building a comprehensive insight into the lived experience of aging women with PCOS to design appropriate management options and raise awareness for the long-term burden of disease risk associated with PCOS.

## Conclusion

In comparing the lived experiences of pre- and post-menopausal women with PCOS, three overarching themes emerged from the semi-structured patient interviews: low health literacy, dissatisfaction with the patient-provider relationship, and a desire for customized resources to manage PCOS-related health issues. The unique perspective of the post-menopausal woman with PCOS brings attention to the need for tailored education and support as women age with this chronic disease. Findings from this mixed-methods study reinforce the need for healthcare providers to be educated and experienced in assessing PCOS and managing this heterogeneous condition beyond reproductive health. In addition, patient-facing education that is tailored to the PCOS woman is needed to support successful and sustainable lifestyle management practices. These needs may be addressed through future development of patient-centered tools (e.g., digital resources or print materials) to increase health literacy in women with PCOS, as well as continuing education for health providers to emphasize the unique challenges and needs of women with PCOS across the lifespan.

## Supplemental Material

sj-docx-2-reh-10.1177_26334941261426089 – Supplemental material for A mixed-method analysis of health literacy and indicators of well-being in women with polycystic ovary syndrome across the lifespan Supplemental material, sj-docx-2-reh-10.1177_26334941261426089 for A mixed-method analysis of health literacy and indicators of well-being in women with polycystic ovary syndrome across the lifespan by Crystal C. Douglas, Ahmar Hashmi, Alexis Mclain and Emily J. Arentson-Lantz in Therapeutic Advances in Reproductive Health

sj-pdf-1-reh-10.1177_26334941261426089 – Supplemental material for A mixed-method analysis of health literacy and indicators of well-being in women with polycystic ovary syndrome across the lifespan Supplemental material, sj-pdf-1-reh-10.1177_26334941261426089 for A mixed-method analysis of health literacy and indicators of well-being in women with polycystic ovary syndrome across the lifespan by Crystal C. Douglas, Ahmar Hashmi, Alexis Mclain and Emily J. Arentson-Lantz in Therapeutic Advances in Reproductive Health

## Declarations

**Ethics approval and consent to participate:** The study protocol was conducted in accordance with the Declaration of Helsinki and approved by The University of Texas Medical Branch’s (UTMB) Institutional Review Board (IRB#23-0011). This study was registered with ClinicalTrials.gov as NCT05769426. All participants provided electronically signed written informed consent to participate.

**Consent for publication:** All participants consented to publication of de-identified data.

**Availability of data and materials:** Data will be made available upon request.
