# Supplementary material for: A mixed-method analysis of health literacy and indicators of well-being in women with polycystic ovary syndrome across the lifespan
Source: Ther Adv Reprod Health. 2026 Feb 22;20:26334941261426089. doi: 10.1177/26334941261426089 (PMC12926541; doi:10.1177/26334941261426089)
Supplement: sj-docx-2-reh-10.1177_26334941261426089 – Supplemental material for A mixed-method analysis of health literacy and indicators of well-being in women with polycystic ovary syndrome across the lifespan [file sj-docx-2-reh-10.1177_26334941261426089.docx]

**Supplemental Table 1:** Semi-Structured Interview Guide used for interviews with pre- and post-menopausal women with PCOS

| **Questions** | |
| --- | --- |
| **1.** | Can you describe your experience in receiving a PCOS diagnosis? |
| **2.** | Can you describe your understanding of the medical condition, PCOS? Currently, what is your primary concern with the diagnosis? |
| **3.** | Can you tell me about the resources you have used in the last 12 months that have provided help with your PCOS journey? And, how did you learn of these resources? |
| **4.** | Are there additional resources you wish you could access to assist in your management of PCOS? |
| **5.** | What challenges have you encountered in managing PCOS in the last 12 months? |
| **6.** | Can you describe your confidence level in your ability to manage PCOS? |
| **7.** | What improvements, if any, do you believe would improve your management of PCOS? |
| **8.** | Is there any additional information that you would like to discuss? |
